# Sex Steroids, Adult Neurogenesis, and Inflammation in CNS Homeostasis, Degeneration, and Repair

**DOI:** 10.3389/fendo.2018.00205

**Published:** 2018-04-30

**Authors:** Tracy A. Larson

**Affiliations:** Department of Biology, University of Virginia, Charlottesville, VA, United States

**Keywords:** apoptosis, astrocyte, microglia, neural homeostasis, neural plasticity, neurodegenerative, neurogenesis, steroidal hormone

## Abstract

Sex steroidal hormones coordinate the development and maintenance of tissue architecture in many organs, including the central nervous systems (CNS). Within the CNS, sex steroids regulate the morphology, physiology, and behavior of a wide variety of neural cells including, but not limited to, neurons, glia, endothelial cells, and immune cells. Sex steroids spatially and temporally control distinct molecular networks, that, in turn modulate neural activity, synaptic plasticity, growth factor expression and function, nutrient exchange, cellular proliferation, and apoptosis. Over the last several decades, it has become increasingly evident that sex steroids, often in conjunction with neuroinflammation, have profound impact on the occurrence and severity of neuropsychiatric and neurodegenerative disorders. Here, I review the foundational discoveries that established the regulatory role of sex steroids in the CNS and highlight recent advances toward elucidating the complex interaction between sex steroids, neuroinflammation, and CNS regeneration through adult neurogenesis. The majority of recent work has focused on neuroinflammatory responses following acute physical damage, chronic degeneration, or pharmacological insult. Few studies directly assess the role of immune cells in regulating adult neurogenesis under healthy, homeostatic conditions. As such, I also introduce tractable, non-traditional models for examining the role of neuroimmune cells in natural neuronal turnover, seasonal plasticity of neural circuits, and extreme CNS regeneration.

## Introduction

Sex steroids coordinate the development and maintenance of male and female reproductive systems, and a multitude of other organ systems, including the digestive, metabolic, skeletal, immune, and nervous systems. Sex steroids are robust regulators of many diseases that, unsurprisingly, occur in organ systems in which steroidal hormones regulate development and homeostasis. Steroidal hormones influence morphology, physiology, and ultimately behavior from the cellular level to the organismal level through a variety of effectors, or classes of sex steroids, their specific receptor types, and several intracellular signaling pathways. In this manner, sex steroids spatially and temporally coordinate vast molecular networks. These networks regulate processes including, but not limited to, cellular proliferation, differentiation, motility, survival, and apoptosis. Specifically within the central nervous system (CNS) sex steroids directly regulate neural activity, nitric oxide signaling, and growth factor expression, among others (1). Through these and other direct processes, sex steroids also secondarily affect the birth and survival of neuronal, glial, and endothelial cells.

Within the context of immune function, sex steroids exert effects on and are one of many effectors of inflammatory cells and immune responses within the CNS. It has become increasingly evident over the last 30 years that the prevalence and severity of neuropsychiatric and neurodegenerative diseases with a neuroinflammatory component are linked to genetic sex [i.e., male versus female (2, 3)]. For example, women are more frequently diagnosed with atypical depression, depression with anxiety, and more severe progression of Alzheimer’s Disease than men [reviewed in Ref. (3)]. As such, recent efforts have focused on understanding the implications of estrogen interactions with the neuroimmune system, primarily in the context of neurodegenerative disease and the possible use of estrogens and inhibitors of estrogen signaling as therapeutics. However, there is mounting evidence that the two other classes of sex steroids, androgens and progestogens, also modulate immune responses. Moreover, all of the sex steroids along with the classically described inflammatory cells have emerged as critical players in maintaining cell and tissue integrity under homeostatic and non-pathological conditions. Thus, understanding the role of all sex steroids, in all sexes, and under non-pathological conditions is paramount for unraveling the complex interactions between the hormonal, specifically sex steroidal, and immune systems, and for ultimately developing therapeutics for maintaining and restoring proper cognitive function.

In this review, I aim to present a comprehensive and integrative view of the independent, antagonistic, and synergistic impacts of sex steroids, the neuroinflammatory system, and neural stem cells (NSCs) for neural homeostasis, neuroprotection, and repair following neural insult. After providing a brief overview on sex steroid biosynthesis and intracellular signaling mechanisms, I discuss the steroidal hormone regulation of neural activity and synaptic plasticity, growth factor expression and responses, and apoptosis. With the intent to bridge three vast and recently converging areas of focus in neuroscience, I first lay a framework by describing the impacts of sex steroids on various neural and inflammatory cell types, including NSCs, neural endothelial cells, microglia, astrocytes, and leukocytes, among others. I then discuss the most recent findings at the intersections of sex steroids, neuroinflammation, and postnatal neurogenesis. I finally present several new models that have great potential to provide novel insights into inflammatory regulation of homeostasis, natural plasticity of neurogenesis, and extreme functional repair and recovery following neural damage and degeneration.

## Sex Steroid Biosynthesis and Signaling Mechanisms

Two major classes of sex steroids, androgens and estrogens, were classically delineated as male- and female-specific hormones as a result of their respective pronounced synthesis in the testes and ovaries and their generalized roles in promoting secondary masculinizing and feminizing effects. Yet, the discovery of extra-gonadal biosynthesis of estrogens from androgens in adipose tissue in 1947 (4) and the birth of the “organizational hypothesis” in 1959 from work that demonstrated testosterone feminizes the CNS of the guinea pig [(5) and reviewed in Ref. (6)] revolutionized the thought and study of sex steroid function. Subsequently, estrogens and androgens have been discovered to be synthesized and exert regulatory effects in multiple organ systems in both males and females.

**Figure 1:**
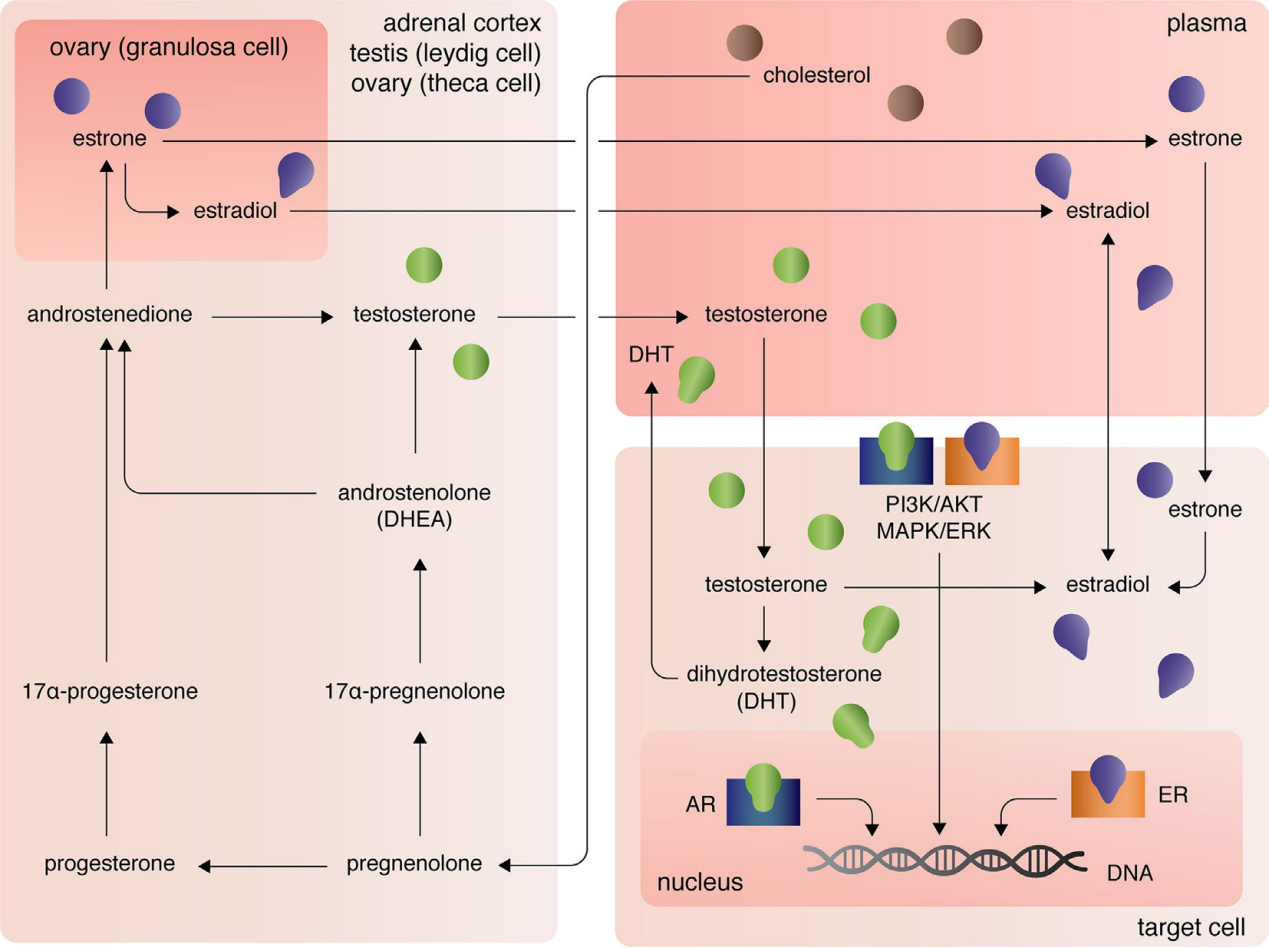
Biosynthesis and localization of sex steroids, with a focus on estrogens and androgens.

The three major types of endogenous estrogens are estrone (E_1_), 17β-estradiol (E_2_), and estriol (E_3_), with E_2_ being the most prevalent and potent form of circulating estrogen. The primary mode of E_2_ biosynthesis occurs *via* the precursor, estrone, which itself is synthesized from androstenedione produced in the theca internal cells of the ovaries in females (Figure 1). The enzyme 5α-reductase, also called aromatase, converts androstenedione to E_1_, which itself can be converted into E_2_ by 17β-hydroxysteroid dehydrogenase (17β-HSD). In both males and females, 17β-HSD can also convert androstenedione into testosterone, which can be converted into E_2_ by aromatase [reviewed in Ref. (7)]. After the discovery of extra-gonadal biosynthesis of E_2_ in adipocytes, evidence of E_2_ biosynthesis has been observed in astrocytes, neurons, and ependymal cells of the brain, osteoblasts, fibroblasts, adrenocortical cells, parietal cells of the intestines, smooth arterial muscle cells, and splenic T cells [reviewed in Ref. (8)].

The androgens testosterone (T) and androstenedione are synthesized primarily by the Leydig cells of the testes (in males) and the zona reticular and zona fasciculate of the adrenal cortex in males and females (Figure 1). Circulating plasma T and androstenedione can be converted to dihydrotestosterone (DHT), a potent androgen receptor ligand, by the enzyme 5α-reductase in target tissues. Likewise, dehydroepiandrosterone (DHEA), also known as androstenolone, is produced in both males and females in the adrenals [reviewed in Ref. (7)]. Circulating DHEA can be converted into both DHT and estrogens in peripheral tissues and itself can bind the different estrogen receptors with varying affinities [for more see Ref. (9)]. As mentioned, T and androstenedione can also be converted to E_2_ by aromatase in both males and females.

A third class of sex steroids, progestins, includes progesterone, which is synthesized from pregnenolone in the corpus luteum of the ovaries and in the adrenal cortex (Figure 1). Progesterone can be converted by 7α-hydroxyprogesterone into androstenedione, which, again, is a precursor of T and E_1_ [reviewed in Ref. (7)]. Extra-gonadal and extra-adrenal production of progesterones has also been observed in the pineal, thalamus, cerebellum, and tectum among other areas of the CNS (10).

At peripheral target sites, all steroid hormones bind nuclear and transmembrane receptors to drive complex, yet precisely tuned cell-type specific signaling responses. Briefly, the estrogens E_1_, E_2_, and E_3_ diffuse across the lipid membrane and bind cytoplasmic estrogen receptors alpha (ERα) and beta [ERβ; for more on affinities, see Ref. (11)]. Ligand-bound ERα and ERβ dimerize, rapidly translocate to the nucleus, and directly regulate transcription of over 3,600 mammalian genes (12). Estrogens can also bind the G-protein-coupled receptors GPER (primarily expressed in adipocytes and intestinal cells), GPER1 (neural and adrenocortical cells), and GPER30 [endothelial cells; reviewed in Ref. (13)]. Estrogen-activation of the GPERs (and likely membrane-associated ERs, as well) initiates PI3K/AKT and MAPK/ERK signaling pathways (13), that in turn coordinate the transcription and translation of whole gene families that regulate cellular processes including, but not limited, to cellular differentiation, proliferation, survival, and apoptosis (14). Similarly, androgens and progesterones exert their functional effects on transcription and translation through intracellular canonical genomic signaling and the non-genomic signaling cascades PI3K/AKT and MAPK/ERK (15, 16). Differential androgen signaling depends on cytoplasmic- versus membrane-targeted isoforms of the androgen receptor gene (16). By contrast progesterone receptors, like estrogen receptors, are encoded by distinct genes and include the intracellular receptors PR-A and PR-B and the membrane progesterone receptor isoforms alpha (mPRα), beta (mPRβ), and gamma (mPRγ) (17). Further, contributing to the complexity of sex steroid receptor signaling mechanisms, unbound estrogen and androgen receptors can bypass activation by their cognate ligands and functionally bind to transcription response elements, albeit with much lower affinity (16).

## Overview of Sex Steroid Functions within the CNS

Through their various metabolites, receptors, receptor isoforms, and intracellular signaling mechanisms, sex steroids regulate vast molecular networks to spatially and temporally coordinate a multitude of processes related to cellular proliferation, motility, differentiation, and survival. Within the CNS, sex steroid signaling directly regulates neural activity, growth factor expression, endothelial cell and NSC proliferation, survival, and apoptosis (15, 18).

### Neural Activity and Synaptic Plasticity

During development sex steroids, especially estrogens, play a pivotal role in arborization, synaptogenesis, and circuit formation (19). For example, the structural, connective, and physiological differences between the male and female brain are a result of gonadal steroid action during development. Regardless of genetically determined sex, morphological, and physiological masculinization of the brain occurs with exposure to E_2_ but not DHT during a restricted developmental window [reviewed in Ref. (6)]. Feminization of the brain and behavior results from a lack of early steroidal hormone exposure (6). One mechanism through which E_2_ affects the development of neural connections is through the modulation of glutamatergic synapse formation. For example, in the developing hippocampus, E_2_ bound ERα stimulates expression of vesicular glutamate transporter 1 and the post-synaptic NMDA receptor, which in turn increases glutamatergic synapse formation of hippocampal neurons (20). For more information on these and other mechanisms driving sexual differentiation of the brain see Ref. (6).

**Figure 2:**
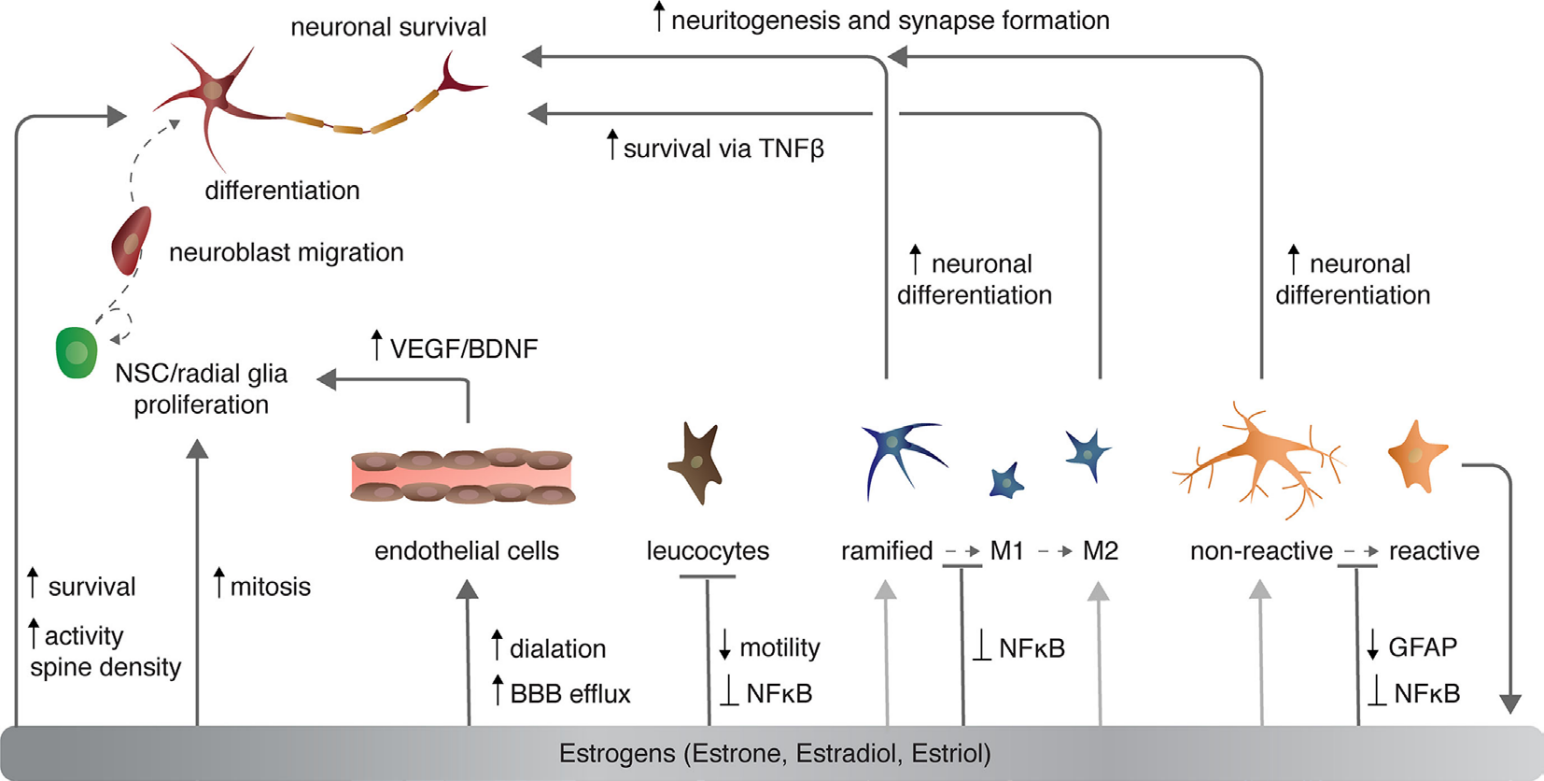
The effects of estrogen on various cell types in the central nervous system. Light gray arrows indicate that estrogens have an effect on neurons via the given cell type to which the arrow points.

Sex steroids also critically regulate neural activity and synaptic plasticity to influence circuit structure and activity in the adult CNS (Figure 2). In hippocampal CA1 pyramidal neurons of adult female rats, E_2_ increases NMDA and AMPA receptor activity, which in turn increase neuronal sensitivity to NMDA (21). This E_2_-mediated enhancement in NMDA sensitivity drives increased dendritic spine density of the pyramidal neurons (21). The influence of progesterone in modulation of dendritic spine formation and neural activity is, however, less clear and complicated by age and prior steroidal hormone exposure. For example, Edwards et al. found that progesterone enhances evoked responses from CA1 field recordings of adult female hippocampal slices (22), whereas Ito et al. observed no effect of progesterone on long-term potentiation in CA1 slices from 4-week-old rats (23). Moreover, progesterone and E_2_ treatment together diminishes the enhanced glutamate-mediated release of intracellular calcium that each hormone has when administered independently (24).

Aside from canonical genomic signaling at the nucleus, sex steroids can rapidly modulate synaptic transmission through changes in spine density and neuron sensitivity within the synaptic process itself. For instance, T and DHT positively regulate spine formation through extranuclear ARs localized within dendritic spines and activation of local MAPK/ERK signaling (25). Local activation of MAPK/ERK drives rapid cytoskeletal and proteomic changes necessary for spinogenesis and plasticity (discussed further below). Similarly, ERα localizes not only within post-synaptic CA1 spines (26) but also pre-synaptic CA3 terminals, where it rapidly promotes long-term depression and spine formation through MAPK signaling (27).

### Growth Factor Regulation

**Figure 3:**
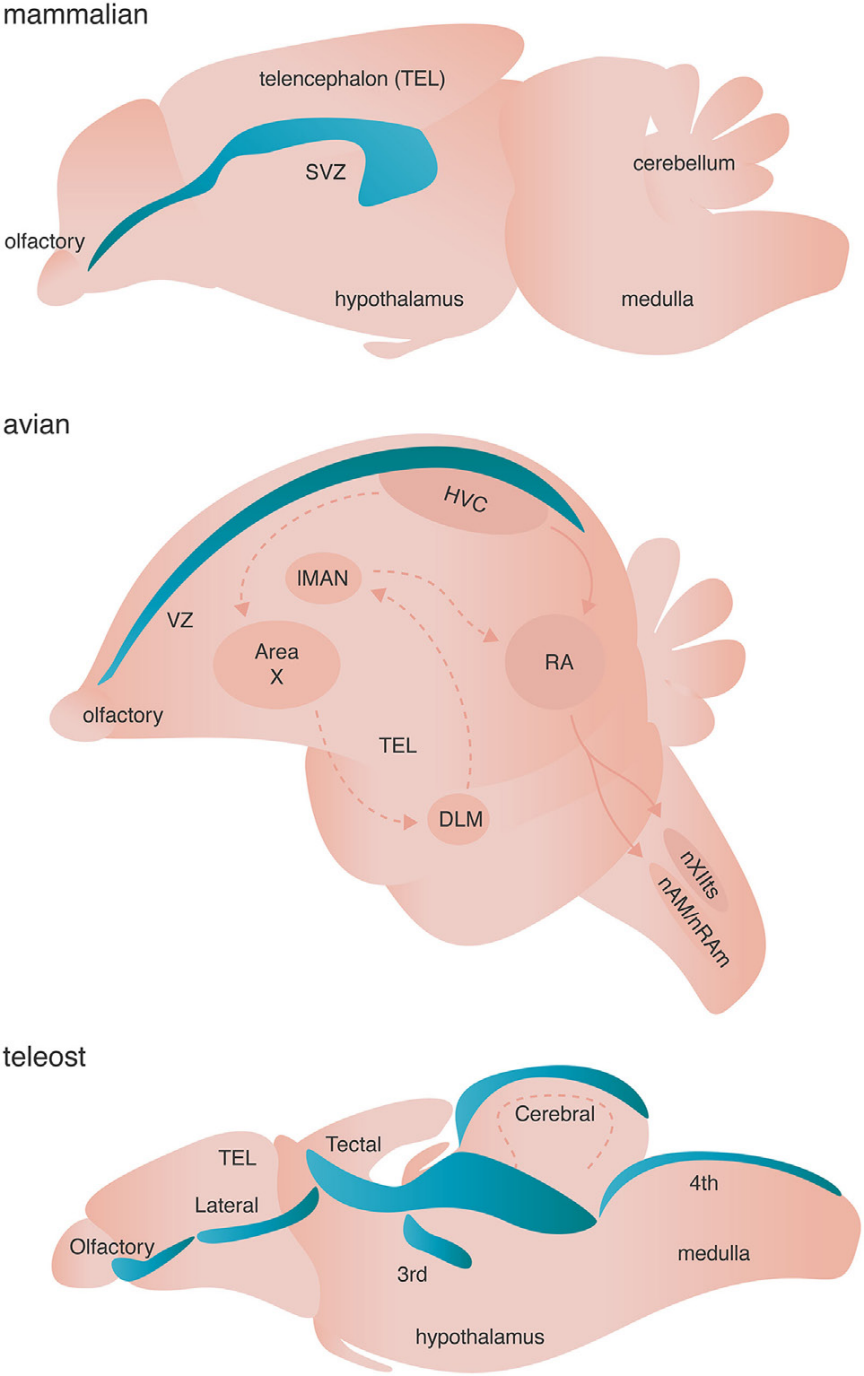
Neurogenic niches in mammals, songbirds, and teleost fishes. Niches are colored blue. Dashed arrows in the avian brain represent the anterior forebrain pathway responsible for song learning, whereas solid arrows in the avian schematic represent the song motor pathway of songbirds. Both pathways are simplified for clarity.

Over 40 years ago, sex steroids, specifically estrogens, were hypothesized to act in conjunction with neurotrophins to induce classic growth factor responses including increased cellular proliferation, differentiation, and survival (28). Subsequently, interactions between sex steroids and neurotrophins have been identified as critical regulators of sex-associated differences in body mass, cardiac function, bone mineral density; hepatic regeneration; tumor formation, and growth; and neural cell function and plasticity [reviewed in Ref. (29)]. Specifically within the CNS, sex steroid receptors co-localize with growth factor receptors including the insulin growth factor (IGF1) receptor IGFR, the low-affinity nerve growth factor receptor p75NTR, and the tropomyosin receptor kinase (trk) family—trkA (nerve growth factor; NGF), trkB (brain-derived neurotrophic factor and neurotrophin 4; BDNF and NT-4), trkC (NT-3). Co-localization occurs within, but is not limited to, the developing mammalian forebrain (30), cerebellum (31), and cortex [*in vitro* (32)], and the telencephalic nucleus HVC [proper name (33)] of the avian song production circuit (Figure 3) among other regions functionally related to the song system (34, 35). Such co-localization of steroidal hormone receptors and neurotrophic receptors drives neurotrophic-dependent processes through one or more of several mechanisms including: (1) convergence on the PI3K/AKT and MAPK/ERK signaling pathways to increase transcription and translation of gene families related to well-characterized neurotrophic processes, (2) autonomous enhancement in expression of neurotrophins and their cognate receptors, and (3) a cascade effect driving non-autonomous expression of neurotrophins.

Sex steroids and growth factors synergistically interact through convergence on the PI3K/AKT and MAPK/ERK signaling pathways to directly and indirectly modulate activity of downstream signaling cascades, transcription factors, and translation machinery. For example, PI3K activation by ligand-bound sex steroid receptors and/or growth factor receptors results in phosphorylation of AKT, which ultimately increases CREB-mediated transcription and ribosomal S6 kinase translation *via* mTOR activation (36). Likewise, both PI3K and MAPK activation increase phosphorylation of GS3Kβ, inhibiting GS3Kβ’s pro-apoptotic effects (37). As eluded to above, convergence on the PI3K and MAPK cascades can occur not only within the neuronal soma but also in pre- and post-synapses to drive rapid local responses to sex steroids and growth factors. Within the nucleus, E_2_ directly regulates transcription through ER activity at estrogen response elements located in the promotor region of several growth factors—VEGF, transforming growth factor alpha (TGF-α), BDNF, NT-4, and NGF [reviewed in Ref. (2)]. Alternatively, within the dendritic spines, sex steroids can bind extranuclear ERs and ARs to enhance local BDNF translation *via* activation of PI3K/AKT and MAPK/ERK (25, 38). This in turn supports cytoskeletal rearrangement of dendritic spines and ultimately long-term potentiation by upregulating: the structural proteins growth-associated protein 43 and microtubule-associated protein 2 [reviewed in Ref. (19)]; the phosphorylation of the actin cleaving protein coffilin (39); and synthesis of essential synaptic proteins such as PSD-95 (40). In this manner, sex steroids and neurotrophins, specifically BDNF, rapidly modulate dendritic spine formation and stabilization to facilitate synaptic and circuit plasticity. At the pre-synaptic terminal, E_2_ also increases retrograde transport of BDNF through a trkB-dependent mechanism in olfactory bulb neurons (41), possibly supporting antagonistic neurotrophic signaling, neuronal competition, and circuit plasticity (42).

Sex steroids can also drive autonomous and non-autonomous up regulation of both self and different neurotrophins and their cognate receptors. To highlight one example of many, in songbirds, testosterone, and specifically its E_2_ metabolite, upregulates the expression of VEGF and the VEGF receptor-R2 in endothelial cells within the highly neurogenic nucleus HVC (Figure 3). VEGF in turn induces expansion of the local microvasculature through a sex steroid-independent increase in endothelial cell mitosis (43). Within 1 week of testosterone treatment not only new and mature endothelial cells, but also neurons and astroglia within HVC, upregulate synthesis of BDNF (43). BDNF is both required for and promotes the addition of adult-born projection neurons into HVC (43, 44), thus facilitating growth of the song control circuit and increased quality of singing behavior [reviewed in Ref. (45)]. This example not only demonstrates the neurotrophic cascade effect of sex steroids acting in concert with growth factors, but also introduces effects on vasculature and NSCs that sex steroids have often in conjunction with activation of growth factor signaling.

### Vasculature

Sex steroids modulate cerebrovasculature function through several mechanisms, of which I will briefly discuss three [for more, see Ref. (46, 47)]: angiogenesis and endothelial cell survival, vascular contractility, and blood–brain barrier (BBB) permeability (Figure 2). Angiogenesis requires proliferation, migration, alignment, and differentiation of endothelial cells. Sex steroids, specifically estrogens, regulate all of these processes. Estradiol directly and indirectly increases the expression of growth factors including VEGF (discussed above) and fibroblast growth factor 2 (FGF2) that in turn increase the proliferation of endothelial cells (48). Estrogens also stimulate endothelial cell adhesion and migration through FGF-mediated increases in expression of surface integrins (49) and the formation of capillary-like structures with an internal lumen *in vitro* (50). Conversely, progesterone suppresses endothelial cell proliferation through nuclear progesterone receptor-mediated arrest of endothelial cells in the G1 phase (51).

Sex steroids also modulate cerebrovascular contractility. Chronic exposure to estrogens decreases vasculature tone and increases nitric oxide-dependent dilation in both males and females (52, 53). Estrogens promote vasodilation through both transcriptional increases in vasodilators including prostacyclin, endothelial nitric oxide synthase (eNOS), among others [reviewed in Ref. (46)], and posttranscriptional mechanisms. For example, E_2_ not only enhances eNOS protein and mRNA expression through nuclear ERα (54, 55), but also eNOS activation through PI3K/AKT-mediated phosphorylation of eNOS at serine 1177 (56, 57). Together, increased production and activation of eNOS promotes nitric oxide production and, consequently, endothelium relaxation.

The BBB is a specialized cerebrovascular structure composed of endothelial cells encased in a basement membrane. The BBB is supported by astrocytic feet, pericytes, and local neurons that collectively form the neurovascular unit [NVU; reviewed in Ref. (47)]. The NVU regulates the influx and efflux of ions, molecules, and cells across the BBB to maintain homeostasis (47). Unique from other endothelial cells, the endothelial cells of the BBB are connected with tight junctions and adherin junctions that restrict the paracellular permeability of the BBB (47). By controlling the flow of ions, the BBB creates a high trans-endothelial electrical resistance, which decreases the ability of polar molecules to penetrate the BBB (58). Transport of substances across the BBB is limited to the passive diffusion of low molecular weight lipophilic molecules, polar nutrients including glucose and amino acids *via* solute carriers, and receptor- and adsorptive-mediated transcytosis of specific high molecular weight molecules such as insulin (59).

Sex steroids modulate the permeability of the BBB by enhancing nutrient and efflux transporter expression. Specifically, estrogens increase endothelial expression of glucose transporter 1, which facilitates 2-deoxy-d-glucose uptake into the BBB endothelial cells and transcytosis (60). Estrogens also modulate efflux transporter expression. Efflux transporters, predominately the adenosine triphosphate (ATP) binding cassette (ABC) transporters, prevent many lipophilic molecules from entering the brain and thus limit exposure to potentially harmful molecules (47). The activity of the ABC transporter sub family G member 2 (ABCG2)—alternatively known as the breast cancer related protein—is controlled by estrogens *via* ERβ (61). In endothelial cells, ligand-bound ERβ reduces phosphorylation of AKT, which subsequently reduces the expression and activity of ABCG2 through ubiquitination and proteosomal degradation (61). The decreased expression of ABCG2 results in not only decreased efflux of harmful molecules out of the brain, including amyloid-β protein (62), but also increased transcytosis of molecules such as tyrosine kinase inhibitors and xenobiotics (e.g., pharmaceuticals).

Although untested to my knowledge, the ability of sex steroids to alter BBB permeability through changes in growth factor expression and non-genomic PI3K/AKT signaling seems likely. The growth factor VEGF increases tight junction permeability *via* VEGFR2-mediated phosphorylation and decreases in the expression of a critical regulatory protein of paracellular permeability, occludin (63). Given the previously discussed E_2_-mediated increases in VEGF expression in endothelial cells, E_2_ likely increases permeability of the BBB *via* VEGF-mediated decreases in occludin expression. Likewise, protein kinase C, an upstream regulator of PI3K/AKT signaling also reduces the expression of another tight junction protein claudin-5 (64). Thus, activation of PKC and PI3K/AKT signaling *via* ligand-bound sex steroid receptors may also decrease claudin-5 expression to increase permeability across the BBB.

Estrogens also repress inflammatory-mediated increases in leukocyte diapedesis, or transmigration, across the BBB. Secreted inflammatory factors including nuclear factor kappa-light-chain-enhancer of activated B cells (NFκB) and interleukin-1 beta (IL1β) increase transcriptional activation of cellular adhesion molecules (CAMs). CAMs mediate leukocyte–endothelial cell interactions are necessary for diapedesis and infiltration of leukocytes into the CNS. E_2_ attenuates the release IL1β by astrocytes, activation of NFκB, and expression of ICAM1 (64), thus decreasing the permeability of the BBB to leukocytes. In this manner, E_2_ is thought to mitigate inflammation following stroke or traumatic brain injury.

### Oligodendrocytes

Oligodendrocytes are type of neuroglia that support and maintain the integrity of axons in the CNS. During early development oligodendrocyte precursors arise from neuroepithelial cells in the ventral ventricular zone (VZ) of the embryonic spinal cord and then migrate to and populate the VZs of the brain (65). As the last cell type to populate the CNS, oligodendrocyte precursor cells (OPCs) migrate into the gray and white matter of the entire embryonic CNS. The migration of OPCs is guided by growth factors including platelet-derived growth factor and FGF, netrins, semaphorins, and the chemokine CXCL1 [reviewed in Ref. (66)]. After migration, OPCs differentiate into other two types of oligodendrocytes: (1) myelin forming oligodendrocytes, which ensheath axons with an approximately 1 *µ*m think myelin sheet and (2) non myelin forming oligodendrocytes, also known as perineuronal or satellite oligodendrocytes, which might play a role in the demyelination of nude neurons [reviewed in Ref. (67)]. Oligodendrocytes not only provide trophic support with production and secretion of glial cell line-derived neurotrophic factor, BDNF, and IGF1, but also electrically insulate axons, promote clustering of sodium channels at nodes of Ranvier, and promote microtubule stability in the axon (66).

Interestingly, myelination of axons by oligodendrocytes is tightly controlled by the neuron rather than a preprogrammed developmental clock within the oligodendrocytes themselves. The primary cue for oligodendrocyte ensheathment of axons is electrical activity of neurons. Electrically active neurons downregulate the polysialylated neural cell adhesion molecule (PSA-NCAM), allowing NCAM–NCAM adhesion required for oligodendrocyte–neuron interactions. Neuron firing also causes release of adenosine and ATP during the action potential. Adenosine and ATP promotes OPC differentiation and stimulation of astrocytes, which in turn secrete leukemia inhibitory factor to induce oligodendrocyte myelination (66). After associating with and receiving signals to ensheath by neurons, oligodendrocytes upregulate transport of myelin proteins transcripts, including proteolipid protein (PLP) and myelin basic protein (MBP), to the wrapping processes. PLP and MBP facilitate ensheathment by assisting with membrane trafficking and vertically coupling the multiple layers of membrane of the sheath, respectively (66).

Oligodendrocytes express both nuclear and non-nuclear ERs and PRs, and as such are directly modulated by sex steroids. During development estradiol promotes myelination in the neonatal rat brain, giving rise to higher numbers of oligodendrocytes in female-derived oligodendrocyte cultures than male-derived cultures (68) and possibly contributing to the well-documented higher incidence of neurological autoimmune diseases in females. More specifically, E_2_, but not progesterone or testosterone, delays the exit of OPCs from the cell cycle, thereby increasing OPC proliferation (68). E_2_ also enhances sheath formation through genomic ER signaling at an estrogen response element for MBP (68, 69) and resulting increases of MBP expression (70). Likewise, progesterone also enhances the formation of MBP and PLP positive membrane sheets through the progesterone receptor, although the precise signaling mechanism is still unknown (68, 71). Progesterone, as well as E_2_, also facilitate remyelination following pathological demyelination in the CNS (72, 73). Given the high incidence of autoimmune disorders like multiple sclerosis (MS) in women, demyelination and subsequent remyelination unsurprisingly involves complex interactions between the immune system and steroidal hormones, and as such, will be discussed further below.

### Postnatal Neurogenesis

Contrary to popular belief, new neurons are generated in the CNS from NSCs and neural progenitor cells (NPCs) throughout the entirety of vertebrates’ lives. Pioneering studies in mammals and songbirds in the 1960s and 1980s provided the first evidence that new neurons arise in the adult brain (74) and that adult-born neurons functionally integrate into pre-existing neural circuits and acquire mature neuronal phenotypes (75). Since the discovery of adult neurogenesis several hypotheses regarding the function of adult-born neurons and neuronal turnover have been proposed. The replacement of older neurons by adult-born neurons has been proposed to enable the learning of new memories (76–78), the maintenance older memories (79, 80), the replacement of over-excited neurons (80), or the replacement of neurons weakened by DNA damage or loss of trophic support (81). Many studies have tested these and other hypotheses, however, no studies have conclusively supported or eliminated any of these possible functions of neuronal turnover (78, 81). Here, I will limit discussion to the complex, yet fine-tuned processes of adult neurogenesis, as broadly defined to include proliferation of NSCs, migration of neuroblasts, and the incorporation and survival of adult-born neurons in functional neural circuits. I will briefly introduce studies that highlight the modulation of adult neurogenesis by sex steroids, as the topic has been extensively reviewed in the past. Although many extrinsic and intrinsic factors such as non-steroidal hormones (e.g., glucocorticoids), morphogens (e.g., Shh, Wnt, BMP), neurotrophins, and neural activity also coordinate the processes of adult neurogenesis, in this review I will primarily focus on the role of the neuroinflammatory cells as a major regulator (below). For exhaustive discussion of these and a broad range of other adult neurogenesis related topics including functional importance, consider consulting Ref. (82).

#### Niches and Proliferation

Adult neurogenesis occurs across all investigated vertebrates (and some invertebrates) with varying spatial localization: addition of adult-born neurons is limited primarily to the olfactory bulb and the hippocampus of mammals, but occurs broadly across the telencephalon of birds, and the telencephalon, diencephalon, optic tectum, cerebellum, and hindbrain of fish [Figure 3 (83)]. True stem cells are defined by two critical characteristics: (1) the ability to self-renew through proliferation and (2) the ability to give rise to multiple cell types through differentiation of progeny. The NSCs of the adult vertebrate brain are self renewing and thought to be multipotent, giving rise to neurons, astrocytes, and oligodendrocytes. Two major models have been proposed to describe the identity of NSC. Briefly, one model posits that non radial glial cells characterized by their expression of sex determining region Y-box 2 (Sox2) are the putative multipotent NSCs, whereas the other model suggests that radial glial cells expressing glial fibrillary acidic protein (GFAP) give rise to adult-born neurons and glia [reviewed in Ref. (84, 85)]. Given that both models are supported by experimental evidence (84, 85) and are not necessarily mutually exclusive, diversity in putative NSCs might represent the spatiotemporal diversity of adult neurogenesis not only across species, but also between neurogenic brain regions within a given species. Alternatively, true stem cells in the adult brain might not exist as a single cell, but rather might represent a whole population of unipotent NPCs, which independently give rise to either neurons, astroglia, or oligodendrocytes.

The dynamic lineages of progeny derived from NSCs, including both lineage-non-restricted NPCs and lineage-restricted NPCs like OPCs, further complicate the identity of putative NSCs in the adult vertebrate brain. For example, in the mammalian brain there are two major neurogenic regions: the ventricular–subventricular zone (V–SVZ) of the lateral ventricles and the subgranular zone (SGZ) of the dentate gyrus (DG). The NSCs of the V–SVZ are GFAP positive radial glia-like B cells. B cells are generally quiescent but can be activated by various signaling factors from other cells in the neurogenic niche and transform into GFAP negative transit-amplifying C cells [for mechanisms see Ref. (85)]. Type C NPCs actively proliferate giving rise to doublecortin (DCX) expressing type A neuroblasts that ultimately populate the olfactory bulb [reviewed in Ref. (84, 86)]. Likewise, the NSCs of the SGZ include type I GFAP expressing radial glial-like cells, which give rise to GFAP negative type II NPCs. The type II NPCs are further divided into two subpopulations: (1) type IIa, which express Mash1 and Sox2 and are highly prolific and (2) type IIb, which express Prox1, NeuroD1, and DCX for early commitment to the neuroblast fate [reviewed in Ref. (84, 86)]. Type IIb neuroblasts ultimately incorporate into the local network in the granular cell layer of the DG.

The conservation of NSCs/NPCs lineage relationships across neurogenic niches within mammals and more generally across vertebrates, suggests a common evolutionary origin for the development of NSC/NPC pools in the adult vertebrate brain. For example, the adult avian brain contains proliferative type B cells in the VZ of the telencephalon that give rise to type A migrating neuroblasts (87). NSCs of the adult zebrafish telencephalon exists as type I GFAP positive cells that are quiescent until transforming into type II GFAP positive radial glial cells. Interestingly, the slowly proliferative type II cells can give rise to both new type I cells through Notch signaling and highly proliferative type III GFAP negative neuroblasts [reviewed in Ref. (88)]. These transit-amplifying type III neuroblasts can continue to proliferate, but can also begin fate specification by turning on PSA-NCAM and the pro-neural gene ascl1 (88). The NSCs/NPCs of the neurogenic niches in the zebrafish cerebellum and optic tectum express the classic NPC marker Sox2 and PCNA, but not GFAP (88).

Although it is generally accepted that adult neurogenesis continuously occurs across all non-human vertebrate lifespans [albeit to a lesser extent with age (89, 90)], the degree to which NSC/NPC proliferation occurs across the human lifespan has become a matter of recent intense debate. In 1998 NPCs in the human brain were first identified in postmortem hippocampal tissue of cancer patients who previously received an injection of the thymidine analog bromodeoxyuridine (91)—a commonly used reagent for labeling mitotic cells. BrdU-labeled cells were identified in the granule cell layer, the SGZ, and the hilus of the DG (91). Given that the average age of the patients examined in this study was 64.4 ± 2.9 years, the authors concluded that adult neurogenesis occurs throughout life in the human DG (91). In 2013 Spalding et al. birth dated hippocampal neurons in humans exposed to nuclear-bomb-test-derived 14C and calculated that around 700 new neurons are generated in the hippocampus per day with a turnover rate of 1.75% annually in nearly one-third of the population of hippocampal neurons (92). Again, the authors concluded that although the majority of hippocampal neurons do not turnover (thus the average age of hippocampal neurons increases with the age of the individual), turnover does indeed continue throughout the entire human lifespan (92). Moreover, in April of 2018 neurogenesis was found to persist in postmortem adult hippocampi of otherwise healthy humans ranging in age from 14 to 79 years (93). The degree to which hippocampal neurogenesis occurs subsequent to the first year of life, however, has recently been challenged. Using postmortem and postoperative samples of human hippocampi, Sorrells et al. reported in March of 2018 that proliferation in the DG peaks at 14 gestational weeks and decreases rapidly by 22 gestational weeks (94). By 7 years of age, nestin, vimentin, or GFAP positive cells with NSC/NPC morphology were not detectable in the SGZ or hilus of the DG, suggesting that the proliferative progenitor pool is near completely, if not entirely, depleted by 7 years of age (94). The apparent contradictions across studies regarding the degree to which adult neurogenesis occurs throughout the human lifespan likely arises from the large diversity in the manner through which the human samples were obtained. Given the difficulty in obtaining human samples in general and the near impossibility of controlling for the plethora of factors that alter adult neurogenesis in these human studies participants, the issue of degree to which NSC/NPC proliferation occurs in humans will likely continue to be debated for some time.

#### Migration

Generally, migration of neuroblasts occurs between one and fourteen days following birth by means of one of three modes—radial, tangential, or undirected wandering migration. In the mammalian CNS, neuroblasts originating from precursors in the SGZ translocate by means of radial migration into layer CA1 of the hippocampus, although typically after tangentially migrating independent of radial glia cells along the local vasculature (95). Alternatively, neuroblasts from the V-SVZ tangentially migrate to the olfactory bulb along chains of PSA-NCAM positive cells forming the rostral migratory stream (96). These PSA-NCAM positive chains consist of a network of differentiated neurons, astrocytes, and tanycytes (97). Migration of neuroblasts along the rostral migratory stream into the olfactory bulb occurs at a rate of nearly 120 µm/h [*in vitro* (98)] and as such generally occurs over 2 days and 2 weeks (99). In the mammalian brain (and other vertebrate brains too), radial glia, microvasculature, and the cells of the PSA-NCAM chains provide trophic support for the migrating neuroblasts through a multitude of factors including, but not limited to, IGF1 and BDNF (95, 100).

In songbirds, a small fraction of the neuroblasts originating from the lateral ventricles migrate mediolaterally along the fibers of radial glia projecting through the telencephalon (101, 102). However, not all neurogenic regions of the avian brain contain radial fibers (102). Rather, neuroblasts translocating to the olfactory bulb migrate tangentially on chains of PSA-NCAM in the avian telencephalon (74). Neuroblasts destined for HVC wander from the VZ without apparent directionality to and within HVC (103).

#### Integration and Survival

Newly generated neuroblasts undergo fairly dramatic attrition before successful incorporation into their respective neural circuits: generally around 50% of neuroblast die via programmed cell death across all neurogenic regions of all vertebrate brains investigated. The “selection process” on neuroblasts is tightly regulated by a plethora of factors, including sex steroids, neurotrophins, and inflammatory cytokines, among many others [for mechanisms see Ref. (82)]. In the DG nearly half of adult-born neurons die within 2 weeks of birth. The neuroblasts that survive the initial culling begin neurite outgrowth with dendrites extending into the molecular layer of the DG and axon extension into the hilus of the DG. By 2 weeks, nascent neurons in the DG begin spinogenesis and the axons begin forming functional connections on CA3 pyramidal neurons in the hilus. By 28 days, adult-born neurons of the DG become fully mature and indistinguishable in morphology, physiology, and behavior from pre-existing mature CA1 neurons [reviewed in Ref. (104)].

After 2–14 days, neuroblast arising from the V–SVZ exit the rostral migratory stream and migrate radially into the olfactory bulb, but not before nearly half of neuroblasts undergo apoptosis (99). Between 15 and 30 days, the persisting olfactory bulb neuroblasts differentiate into one of two types of local interneurons. The vast majority, around 95%, differentiate into GABAergic granule cells, whereas the remaining 5% differentiate into periglomerular neurons expressing either GABA, or dopamine, or both (99).

As is true with the other populations of new neurons in the mammalian brain, nearly 50% of all cells born from NSCs/NPCs undergo apoptosis during migration to their final destination in the avian brain as well (105). The majority of investigations characterizing the fate of neuroblasts in the avian brain originate from studies in the song control circuit, although recently studies have also examined adult neurogenesis in the avain hippocampus (106). Within the song control circuit nascent HVC projection neurons begin to express neuronal markers and form synapses on targets up to 4 mm away in the robust nucleus of archopallium (RA) as early as 2 weeks of age (105). By 28 days to 8 months, most new HVC neurons that survive the initial stages of culling form synapses onto RA neurons (105, 107). These new HVC to RA projection neurons persist for months (108) to years (109, 110), depending on their time of birth and the presence of sex steroids [reviewed in Ref. (81)].

#### Sex Steroidal Modulation

Sex steroids have complex roles in regulating adult neurogenesis, as broadly defined. Both E_2_ and progesterone treatment independently increase mitosis of NSCs/NPCs [Figure 2 (111, 112)]. However, as is the case with modulation of dendritic spine density (above), progesterone and E_2_ reduce the effect of each steroidal hormone when administered together (112). Several studies have shown that natural seasonal plasticity of androgens and castration with supplementation of exogenous androgens enhance the survival of new neurons in both mammals and birds [reviewed in Ref. (3, 81)]. More specifically, the androgen metabolite DHT consistently promotes new neuron survival, whereas the metabolite E_2_ has mixed effects, depending on the mammalian species examined, the sex of the animal, and timing and duration of E_2_ exposure [reviewed in Ref. (3)]. Alternatively, in HVC of both male and female birds, both DHT and E_2_ act independently and synergistically to enhance addition and survival of new neurons (113, 114). Sex steroids exert these pro-neurogenic effects by promoting increased DNA synthesis, transcription, and growth factor signaling and processes [for more see Ref. (3, 15, 81)].

### Apoptosis

As discussed throughout, sex steroids generally promote the survival of new and mature neurons. Estrogens, however, have also been proposed to potentiate excitotoxic neuronal death, especially following pathological damage (2). This working hypothesis is supported by the increased sensitivity and excitability of neurons exposed to E_2_ *via* estrogen-mediated increases in NMDA and AMPA receptor activity (discussed above). Increased firing of neurons, specifically through NMDA signaling in turn rapidly increases E_2_ synthesis (115), and thus can drive a feed-forward cycle that could result in excitotoxic cell death. In this context, estrogens facilitate seizure activity in both animal models and humans (116, 117) through the initiation of an NMDA–E_2_ positive-feedback loop (118). Clearly, there exists a fine balance between the beneficial and harmful effects of sex steroids in the maintenance of homeostasis and on neuron survival. And so, careful analysis of the spatiotemporal expression of sex steroids across the sexes and the effects of interactions between the classes of sex steroids on cell proliferation, behavior, and survival is critical for garnering a more thorough understanding of the basic biology and potential restorative value of sex steroids.

## Sex Steroid Action on Neuroinflammatory Cells

Sex steroids play a pivotal role in the development and function of the immune system through both rapid and long-term mechanisms. Besides regulating the proliferation, migration, and differentiation of immune cells, sex steroids are generally neuroprotective under inflammatory conditions. The anti-inflammatory potential of sex steroids, specifically estrogens, originates from observations that the drop in steroidal hormones associated with menopause increases the incidence of neuroinflammatory associated neuropathologies [reviewed in Ref. (119)]. Subsequently, estrogens, and progesterones to a more limited degree, have been demonstrated to exert their generally protective regulatory action through direct and indirect regulation of immune cell morphology and behavior.

### Microglia

**Figure 4:**
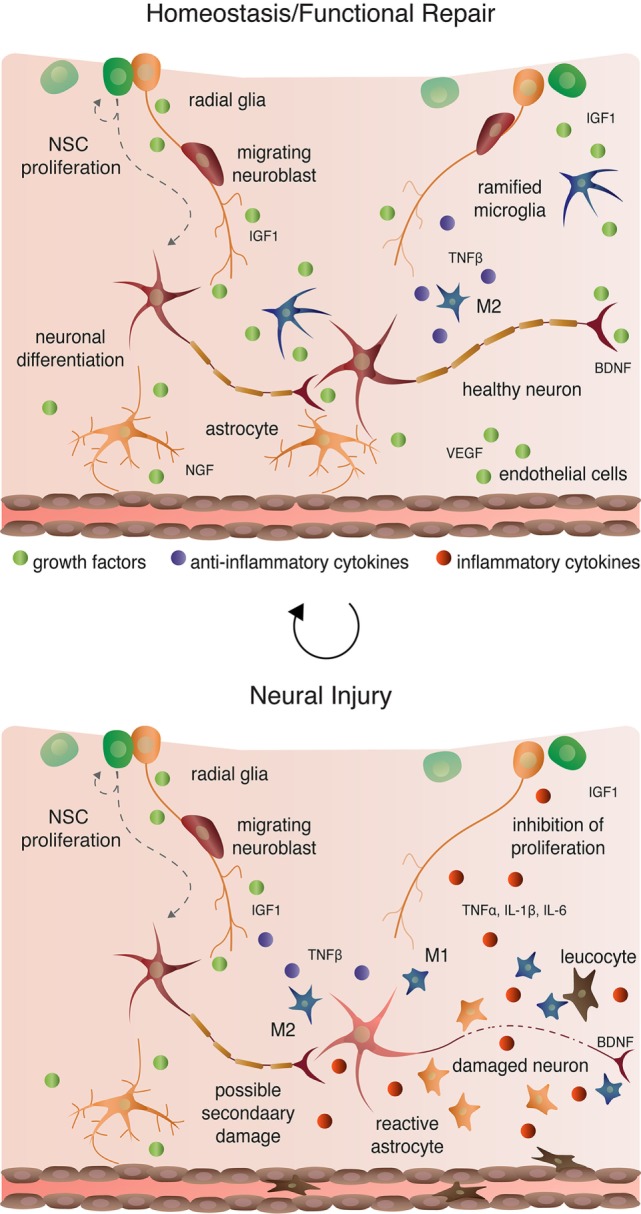
Cellular interactions in homeostatic, injury-induced, and reparative conditions.

Microglia are macrophage-like cells that originate from myeloid precursors during early development and migrate to and take up permanent residence in the developing CNS (120). The primary role of microglia is to continuously survey the microenvironment, remove cell debris and pathogens, and activate reactive inflammation. Microglia have both protective and destructive impacts in the CNS that largely depend on their state of activation (Figure 4). In the un-challenged, healthy brain “resting” or ramified microglia are highly motile (121) using their branches for surveilling their territorial domains for excess neural cells and synapses to phagocytose (122). Microglia remain in the resting or non-activated state through inhibitory signals from the neurons on which the microglial processes directly reside (123). The loss of connection between microglia and neurons, for example during neuronal death, accelerates the activation and response of microglia (123). With neural damage or loss of homeostasis, microglia become polarized to one of two states: M1 and M2 [mechanisms reviewed in Ref. (124)]. Inflammatory M1 microglia induce classical, and often harmful, cytotoxic responses through enhanced antigen presentation and the release of cytotoxic mediators, including NO, tumor necrosis factor alpha (TNFα), IL-1β, prostaglandin, and reactive oxygen species following activation by cytokines, interferons, and endotoxins [reviewed in Ref. (125)]. M1 microglia can molecularly switch to M2 microglia [reviewed in Ref. (124)], which phagocytose dying neurons and promote neurite outgrowth, oligodendrocyte fate specification, and angiogenesis following neuronal death (126–128). The switching between cell types and the balance between protective and restrictive outcomes depends on regulatory molecules in the local environment, including sex steroids, among many others [reviewed in Ref. (124, 125, 129)].

Administration of sex steroids before or after insult generally reduces infarct volume following middle cerebral artery occlusion [MCAO; reviewed in Ref. (129)], with evidence that this neuroprotective effect of sex steroids is mediated in part through microglia. For example, administration of E_2_ and progesterone reduces the attraction of microglia (and leukocytes) to the MCAO-induced penumbra in both male and female rats (130). Moreover, the typically observed pro-inflammatory cytokines CCL2, CCL5, and IL-6 are lower in the penumbra following E_2_ and progesterone treatment, suggesting that activation of local microglia is also suppressed by E_2_ and progesterone (130).

Likewise, E_2_ disrupts microglia activation following stimulation with lipopolysaccharide (LPS)—a lipoglycan endotoxin isolated from the outer membrane of Gram-negative bacteria and commonly used to mimic CNS infection and induce microglia activation (131). Typically, LPS activates the innate immune receptor toll-like 4 [TLR4 (132)] to induce microglia activation *via* MAPK phosphorylation and NFκB nuclear translocation (133, 134). In turn, microglia stimulated by LPS increase expression of pro-inflammatory cytokines (e.g., TNFα), cyclooxygenase-2), and NOS (135). E_2_ activation of microglial ERα, but not ERβ, inhibits the LPS-induced translocation of NFκB family proteins to the nucleus and, consequently, reduces NFκB-dependent transcriptional activity and the resultant inflammatory response [Figure 2 and 4 (136)]. Further supporting a role for E_2_–ERα in microglia reactivity, exogenous systemic supplementation of E_2_ prevents LPS-induced microglial activation and expression of neuroinflammatory mediators, specifically, metalloproteinase 9, and complement C3 receptor in mice (137). However, this impact of exogenous E_2_ on microglia reactivity is lost in ERα-null mice (137).

The effects of steroidal hormones, however, might not be universal across the sexes: in cultured microglia isolated from neonatal hippocampus of male and female rats, E_2_ administration following LPS stimulation of the *ex vivo* microglia attenuates IL-1β mRNA upregulation in male but not female microglia (138). The anti- and pro-inflammatory impact of E_2_ on male and female neonatal microglia, respectively, indicates that E_2_ drives differential microglia responses in males and females during development likely contributes to sexual differentiation of the CNS (139) and that the differential impact of E_2_ across the sexes have effects beyond development. Given the above evidence that E_2_ plays pro-neuroprotective and anti-inflammatory roles in the adult female CNS, additional studies directly testing the role of E_2_ and the other sex steroids to determine when the switch between E_2_-dependent pro- and anti-inflammatory effects occurs in females are necessary.

### Astroglia

Astroglia, a sub-type of macroglial cells, comprise nine biochemically and developmentally distinct forms including radial, fibrous, protoplasmic, among others (140). As discussed above, radial astroglia (more commonly referred to simply as radial glia) are organized in planes perpendicular to the ventricle with one of their two projections extended toward the ventricle and the other deep into the gray matter. As such, radial glia structurally and trophically support migrating neuroblasts and, in doing so, play an extensive role in neural development and adult neurogenesis (Figure 2 and 4). Fibrous astroglia reside along white matter tracts and exhibit long unbranched fiber-like process, whereas protoplasmic astroglia are found throughout gray matter and are characterized by many fine branching processes uniformly distributed around the soma in the classic stellate pattern. Both fibrous and protoplasmic astrocytes establish highly organized territorial domains with their processes, one of which, the “end foot” contacts an intraparenchymal blood vessel whereas the other processes extend into and survey the surrounding microenvironment. In this manner, all astrocytes bi-directionally connect nutrient sources (e.g., cerebrospinal fluid and blood) to nearby glia, neuropil, and synapses, allowing for dynamic responses to microenvironmental changes [reviewed in Ref. (141)].

Under healthy physiological conditions, astrocytes maintain homeostasis and play key specialized roles in the CNS [reviewed in Ref. (142)]. For example, astrocytes not only facilitate high frequency neuronal firing through buffering of local pH (143), uptake of interstitial K+ (144) and glutamate recycling (145), but also modulate neural activity and the propagation of action potentials through the release of glutamate, GABA and calcium, among other “gliotransmitters” (141, 146). Astrocytes also supply energy and nutrients to cells of the CNS through several mechanisms, including, but not limited to: (1) rapid vasodilation during neural activity to increase the availability of oxygen and glucose (147), (2) storage and release of glycogen [reviewed in Ref. (148)], and (3) production, secretion, and distribution of cholesterols that cannot cross the BBB (149). And finally, under normal physiological conditions, non-reactive astrocytes, like ramified microglia, regulate the formation and pruning of synapses (150, 151).

Aside from maintaining stable physiological conditions, astroglia also perform immunological functions similar to CNS-resident microglia and macrophages (Figure 2 and 4). In response to neural damage, astroglia become reactive and undergo morphological and behavioral changes characterized by the upregulation of intermediate filament proteins including vimentin and GFAP, which facilitate reactive retraction of the astrocytic perisynaptic processes (152). As with microglia, the morphological and behavioral changes associated with reactive astrogliosis vary along a continuous spectrum dependent on the severity and the spatiotemporal extent of neural damage. For example, with “mild to moderate” reactive astrogliosis, GFAP upregulation and hypertrophy of perisynaptic processes occurs, but reactive astrocytes maintain their territorial domains and do not proliferate (141). “Severe” reactive astrogliosis occurs as either “diffuse” or “with compact glial scar formation” in response to severe focal lesions, infection, or other chronic neurodegeneration (141). Both severe forms are characterized by upregulation of GFAP, processes hypertrophy, extensive intermingling of territorial domains, cytokine release and leukocyte, microglia, and T-cell recruitment, astrocytic proliferation, and reorganization of tissue architecture [reviewed in Ref. (141, 153)]. Moreover, the increased GFAP expression by astrocytes during reactive astrogliosis suppresses neurite outgrowth *in vitro* (154) and axonal regeneration *in vivo* (155). Severe diffuse astrogliosis progresses to compact glial scar formation as a means to cordon off severe necrosis and leukocyte infiltration and to prevent further neural damage (156). This continuum of reactivity highlights both the beneficial roles of astroglia in the acute stages after neural damage and their detrimental effects on regeneration in the context of chronic neural damage.

Not surprisingly, sex steroids modulate the behavior of astroglia under both normal physiological and pathogenic conditions. In the developing and healthy brain, E_2_ regulates astrocytic expression and secretion of various molecules involved in neurotransmitter transport, mitochondria respiration, steroidal hormone synthesis, and neuritogenesis [for more see Ref. (157)]. Under pathological conditions, E_2_ decreases astrocyte proliferation both *in vitro* and *in vivo* in addition to reducing cytokine secretion and glial scar formation (158–160). The modulation of the astrogliotic response by E_2_ begins at the morphological activation of astrocytes: E_2_ decreases the expression of GFAP and vimentin after injury-induced neuronal loss, ultimately preventing reactive astrogliosis [Figure 2 (159, 161)]. Interestingly, E_2_ stimulates GFAP expression in olfactory bulb astrocytes (162) and increases astrocytic proliferation in white matter (163), suggesting regional and astrocyte-type differences in response to sex steroids and possibly even impacts on neuroprotection.

Generally, sex steroids suppress astrocytic secretion of pro-inflammatory molecules and the subsequent recruitment of additional inflammatory cells. For instance, as with microglial activation, E_2_ prevents LPS stimulated translocation of NFκB to the nucleus and production of TNFα, NO, IL-1β, and IL-6 in astrocytes (164–166). As mentioned above, E_2_ suppression of NFκB in astrocytes also results in decreased astrocytic expression of ICAM1 (64), a CAMs that mediates leukocyte–endothelial cell interactions necessary for transmigration of leukocytes across the BBB into the CNS. In this manner, E_2_ synthesis and secretion by astrocytes may decrease the permeability of the BBB to leukocytes and contribute to the mitigation of inflammation and glial scaring following neural damage.

Neuronal injury within the mammalian and avian CNS routinely enhances the expression and activity of aromatase (167, 168), which, consequently, permits increases in local estrogen synthesis and confers neuroprotection [Figure 2 (169)]. Important to understanding mechanisms of injury-induced aromatase activity, upregulation of aromatase occurs in and around the lesion site regardless of the whether or not the affected brain region produces aromatase under normal physiological conditions (170, 171). In fact, this upregulation of aromatase occurs specifically within the locally activated astrocytes. These astrocytes in turn synthesize and secrete estradiol to decrease local neuronal apoptosis (169), as well as likely inducing nearby proliferation of NSCs and migration of neuroblasts to the region of damage [(discussed above and below (172)].

### Macrophages, Neutrophils, Monocytes, Dendritic Cells (DCs), and Lymphocytes

In addition to microglia and macroglia, sex steroids modulate inflammation through other inflammatory cells as well. CNS-resident macrophages, neutrophils, non-proliferative monocytes, antigen presenting myeloid cells (i.e., DCs), and lymphocytes (i.e., T-, B-, and natural killer cells) continuously surveil the CNS, rapidly infiltrate sites of injury to clear dead cells and pathogens, recruit additional inflammatory cells for tissue repair, and in some contexts, provide protection from similar future insults. Generally, macrophages, monocytes, and neutrophils minimize inflammation through the phagocytosis of dead cells and debris and the release of cytokines and chemokines to recruit additional leukocytes (e.g., monocytes, neutrophils, etc.) to the CNS and site of injury [reviewed in Ref. (173)]. Another phagocyte, the DC, serves as a sentinel by sensing the local environment through continuous and regular endocytosis. Depending on the environmental signals perceived, DCs dictate the type of inflammatory response initiated through differential secretion of cytokines, some of which promote the differentiation of activated T cells, reviewed in Ref. (174). Differentiated T cells can in turn secrete cytokines, stimulate the activation other cell types (e.g., DCs, macrophages, B cells, etc.), and initiate cytotoxic “killer” functions (174). Together, the degree of collaboration of different inflammatory cell types promotes a unique step-wise program and cytokine profile dictating the impact of the inflammatory response. Although initially beneficial, extensive or overreactive activation and recruitment of leukocytes, DCs, and lymphocytes can push the inflammatory response beyond beneficial toward detrimental in terms of neuroprotection and repair (175).

The majority of evidence supporting a role for sex steroids in regulating these other inflammatory cells originates with male-female differences in insult-responsive cell numbers, with a few exceptions [reviewed in Ref. (173)]. For instance, resident macrophages have higher TLR expression and phagocytic activity in females than males, which possibly contributes to the greater response efficiency and survival of females during sepsis (176). Likewise, plasmic neutrophil counts correlate with systemic estrogen levels (177). Although vastly understudied, the mechanistic role of estrogens in regulating leukocytes, lymphocytes, and DCs is becoming more evident. In addition to decreasing NκFB nuclear translocation and TNFα, IL-1β, and IL-6 expression in microglia and astrocytes, E_2_ regulates macrophages and monocytes stimulated with LPS or other neural trauma through these same mechanisms [Figure 2 (178, 179)]. Neutrophils decrease IL-1β and IL-6 secretion following injury to the carotid arteries when exposed to E_2_ (180). Estrogens also regulate the motility of monocytes and neutrophils: E_2_ not only decreases transcytosis of leukocytes across the BBB through changes in BBB permeability (discussed above), but also modulates leukocyte migration through direct action on the leukocytes themselves. Specifically, E_2_ decreases CXCR2, a chemokine receptor that mediates monocyte and neutrophil adhesion and chemotaxis, following cytokine stimulation or injury (180–182). As such, E_2_ suppression of leukocyte migration across the BBB and within the CNS may restrict recruitment of inflammatory cells and, in turn severe inflammatory responses to promote sex steroid dependent neuroprotection.

Estrogens also regulate myeloid cell and lymphocyte differentiation and function, and thus DCs and T cells are involved in mediating the neuroprotective effects of estrogens. For instance, estrogen signaling *via* ERα is necessary for DC differentiation and modulates the acquisition of DC effector functions (183). The estrogen estriol (E_3_) promotes immune self-tolerance functions of DCs through increased expression of anti-inflammatory cytokines IL-10 and TGF-β and decreased expression of pro-inflammatory cytokines IL-12 and IL-6 (183). Likewise, E_2_ promotes the development and function of regulatory T cells, which participate in the maintenance of self-tolerance and, when aberrant, autoimmune disorders through upregulation of the transcriptional factor FoxP3 within T cells (184). Together these data provide the beginnings of a mechanistic understanding for the well-known female sex bias in the development of neurological autoimmune disorders and psychiatric disease [for more, see Ref. (185)]. Future efforts examining the influence of steroidal hormones on myeloid cell and lymphocyte physiology and impacts are undoubtably justified.

## Integration of Sex Steroids, Neurogenesis, and Neuroinflammation

The first conclusive evidence that inflammation impacts adult neurogenesis came in 2003 from a study showing that a 4-week infusion of LPS into the hippocampus decreased NSC proliferation (186). The same year another study demonstrated that systemic inflammation induced through intraperitoneal injection of LPS similarly decreased NSC proliferation (187). Moreover, the LPS-mediated impairment of adult neurogenesis was ameliorated by the administration of inhibitors of microglia activation, minocycline and indomethacin, suggesting microglia were likely regulating NSC behavior (186, 187). Given the 4-week time course of LPS infusion, the observed decrease in NSC proliferation could have resulted from increased NSC apoptosis (122) and reduction of neuroblast incorporation into circuits (188). Yet, subsequent studies have confirmed that LPS promotes a neuroinflammatory response, including depression of NSC proliferation, through direct stimulation of astrocytes and microglia in addition to indirect recruitment of leukocytes, specifically monocytes and neutrophils (189, 190).

Recent studies highlight the dynamic role of inflammatory cells, their interactions, and their responses in modulating adult neurogenesis and the regenerative capabilities of the CNS. Most of such efforts have focused on neuroinflammatory responses to physical damage, such as ischemia and traumatic brain injury, or pharmacological insult, including treatment with LPS or pro-inflammatory cytokines and chemokines. Currently, few studies have directly assessed the role of immune cells in regulating adult neurogenesis, as broadly defined here to include NSC proliferation, neuroblast migration, and nascent neuronal differentiation and survival, under healthy, homeostatic conditions. Below, I briefly discuss advances in understanding the damage-induced inflammatory response on NSC and their nascent progeny after first highlighting the few studies that have addressed the impact of non-reactive and alternatively activated (i.e., M2) microglia on neurogenesis.

### Regulation of Adult Neurogenesis by Microglia

Ramified, unchallenged microglia play a pivotal role in homeostasis not only in the parenchyma (above) but also in the neurogenic niches of the CNS. Ramified microglia instruct neuronal differentiation of neuroblasts through the secretion of a still unknown factor [Figures 2 and 4; (191)] and facilitates clearance by phagocytosis of the ~50% of neuroblasts that do not differentiate and functionally incorporate into circuits (122). Likewise, alternatively activated M2 microglia promote pro-neurogenic effects through the release of anti-inflammatory cytokines including IL-4, IL-10, and TGFβ; tpysinogen, a precursor of the enzyme trypsin; and growth factor such as IGF1 and BDNF [Figures 2 and 4; reviewed in Ref. (124)]. For instance, chronic elevation of microglial TGFβ enhances the survival of proliferative NSC and the differentiation of progeny into the neuronal (as opposed to glial) lineage (192). Likewise, microglia stimulated with IL-4 or IL-10 enhance NPC proliferation, perhaps through the upregulation of IGF1 production following anti-inflammatory cytokine stimulation (193, 194).

Reactive M1 microglia also have immediate beneficial impacts on recovery from neural insult, as they facilitate the initial clearance of debris. Typically, however, in the context of neurogenic repair following clearance of injury- or pathogen-induced neuronal death, M1 microglia have detrimental effects on neurogenesis (Figure 4). M1 microglia secretion of pro-inflammatory cytokines, including TNFα, IL-1β, IL-6, and INF-γ (195), directly impair NSC proliferation, suggesting that the NSCs themselves are capable of detecting and responding to certain cytokines. Besides modulating NSC proliferation, these cytokines impair other processes of adult neurogenesis (as broadly defined) from the migration and differentiation of neuroblasts to final incorporation and survival of new neurons. For instance, IL-1β not only decreases NSC proliferation but also impairs the survival of neuroblasts and hinders neuronal differentiation (196). Although not tested directly as an output of microglia, administration of TNFα, a secreted factor of M1 microglia, promotes the apoptosis of NSC (197) and increases the proportion of daughter cells that differentiate into the astrocytic as opposed to neuronal lineage (198). Likewise, IL-6 reduces NPC proliferation through promotion of progenitor differentiation, albeit into neurons (199).

The differential beneficial and detrimental impacts of microglia underscore the need to further characterize microglial behaviors in healthy and neurodegenerative conditions, and to elucidate mechanisms driving molecular and behavioral switches between states of microglial activation. Additional, carefully controlled studies will also be important for deciphering upstream and downstream regulatory mechanisms of cytokines, growth factors, and other signaling factors: research efforts should be made to disentangle the beneficial versus detrimental impacts of microglia on themselves, other inflammatory cells (astrocytes, leukocytes, DCs, T cells), and CNS-resident cells (endothelial cells of the parenchymal vasculature and BBB, neurons).

### Astroglia, Aromatase, and Adult Neurogenesis

As mentioned above, astroglia can synthesize and secrete E_2_. In songbirds, the number of newly generated cells positively correlates with the number of precursor radial glial cell processes expressing aromatase (200). Likewise, the degree of neurogenesis also correlates positively with the number of aromatase expressing non-precursor astrocytes, and this correlation is abolished with ovariectomy and administration of aromatase inhibitors (172, 201). Non-precursor astrocytes also actively regulate the differentiation and survival of newly generated neurons (Figures 2 and 4). For instance, astrocytic expression of ephrin-B2 induces neuronal differentiation *via* EphB4 receptors on NSCs and subsequent activation of Wnt signaling (202). Moreover, astrocytes facilitate dendritic spine maturation and synapse formation of adult-born neurons through vesicular release of d-serine, a co-agonist (along with glycine) of the NMDA receptor expressed by new neurons (203). However, very few studies have directly tested roles for activated, immunogenic astroglia in regulating NSC proliferation, neuroblast migration and differentiation, or neuronal incorporation and survival. Given the similarities in activation responses between microglia and astroglia, one could easily deduce that if astroglia directly regulate NSCs, then the initial astrogliotic response following injury would likely promote neurogenesis, whereas chronic astrogliosis likely hinders NSC proliferation and new neuron differentiation and survival.

### Regulation of Myelination Through Sex Steroid Impacts on Immune Cells

Sex steroids also enhance the myelination of new neurons and the remyelination of mature neurons following pathological demyelination. As discussed above, under non-pathological conditions sex steroids directly promote the proliferation of OPCs, differentiation of new oligodendrocytes and stimulate oligodendrocyte activities including sheath synthesis and wrapping of axons. The impact of sex steroids on oligodendrocyte behavior also occurs indirectly through immune cell-mediated interactions. For example, the CNS autoimmune disorder MS is more prevalent in women than men. Moreover, the characteristic relapsing and progressive sclerotic plaques and demyelination of MS can be alleviated by treatment with sex steroidal hormones (204). In a murine model of MS administration of the copper chelator cuprizone induces toxic demyelination, which is lessened in severity by treatment with progesterone (73). Likewise, treatment with E_2_ following cuprizone administration also ameliorates demyelination and promotes myelin repair (163). Progesterone and E_2_ both independently induce IGF1 expression in astrocytes, which in turn promotes the proliferation of OPCs and the differentiation of progeny into myelinating oligodendrocytes (163, 205). The beneficial impact of estrogens is, at least in part, mediated through ERα-dependent signaling in reactive astroglia. Treatment of experimental autoimmune encephalomyelitis (EAE) mice, another model for MS, with the selective ERα agonist PLP peptide 139–151 (PPT), decreases the production of TNFα, interferon-γ, and IL-6 in addition to decreasing the recruitment of macrophages and T cells and EAE-associated demyelination (206–208). Conditional gene deletions of ERα in astroglia, but not neurons, reversed the effect of PPT treatment on macrophage and T cell recruitment and prevented estrogen-mediated attenuation of gliosis and axonal degeneration in EAE mice (208). The ERβ agonist WAY-202041 has minimal reported effects on EAE-induced cytokine production (206). Interestingly, however, ERβ, but not ERα, is expressed in microglia. Treatment of EAE mice with the selective ERβ agonist LY3201 promotes microglia to retain their ramified morphology and, as such, reduces microglia NFκB activation and iNOS expression (209).

Microglia and astroglia, in addition to other immune cell types, facilitate not only debris removal following demyelination, axonal damage, and neuronal death, but also neurogenesis, gliogenesis, nascent cell fate specification, arbor and synapse maturation, and nude axonal ensheathment. Although sex steroids clearly modulate the behavior and impact of immune cells, gaps in our understanding of the complex interactions between immune cells, neural cells, and sex hormones still exist. Thus, directly testing the relative impact of reactive astrogliosis and activated microglia on adult neurogenesis and gliogenesis will be essential for understanding not only the unique roles of micro- and macroglia during regeneration but also the manner through which astrocytic and microglial responses integrate into the larger orchestra of neuroinflammation.

## Future Directions: Emerging Models of Neuroinflammation and Regeneration

The role of inflammation in neurocognitive disorders and employment of sex steroidal and anti-inflammatory therapeutics has been discussed extensively in past reviews. So instead, here, I limit the discussion to emerging models of neuroinflammation and regeneration. For more information on the role of inflammation in relatively well-characterized psychiatric and neurodegenerative disorders and classes of therapeutics see Table 1.

**Table 1:** Neurological disorders with known inflammatory component and therapeutics.

**Psychiatric disorders**	
Depression	(119, 210)
Schizophrenia	(119)
Autism	(211, 212)
**Neurodegernative disorders**
Multiple sclerosis	(119, 213)
Amyotrophic lateral sclerosis	(119)
Parkinson’s disease	(119, 214, 215)
Alzheimer’s disease	(15, 119)
**Traumatic injury**
Cerebral ischemia	(119, 216)
Aging	(3, 210)
**Therapeutics**
Selective estrogen receptor modulators	(15, 217)
Blood–brain barrier	(1, 46, 47)
Macrophage delivery	(218)
Mesenchymal stem cells	(216, 219)
Neural stem cells	(216, 219)

### Neurological Diseases With Recently Discovered Immune Component

The reemergence of fetal microcephaly as a result of maternal Zika viral infection has recently leapt onto the main stage as a neurodevelopmental disorder with an underlying neuroimmune component. This is not to suggest that Zika virus (ZIKV) is the only neurological disease with developing evidence for an inflammatory component, but it certainly of recent piqued interest, and thus, I will briefly discuss ZIKV-induced microcephaly here. Following the detection of ZIKV in the fetal brain (220) and establishment of a causal relationship between ZIKV infection and microcephaly (221) in 2016, ZIKV was discovered to infect NPCs and attenuate their growth (222). Specifically within radial glia progenitor cells, ZIKV reduces proliferation and promotes premature differentiation through ZIKV protein NS2A destabilization of the adherens junction complex, and as a result, mis-scaffolding of the radial processes (223). Two other ZIKV proteins, NS4A and NS4B, cooperatively inhibit AKT and mTOR signaling in NSCs, leading to decreased proliferation and increased autophogy of the NSC (224). ZIKV also infects microglia of human fetal brain, rendering the microglia reactive. ZIKV-infected microglia secrete high levels of pro-inflammatory factors TNF-α, IL-1β, IL-6, among several others (225), which likely further decreases embryonic neurogenesis through mechanisms discussed above. Cleary, additional studies determining the mechanisms of ZIKV’s negative impact on neurogenesis and neuroinflammation are warranted and these mechanisms will certainly be taken into consideration during the development of therapeutics and vaccines. Given that ZIKV infects both stem cells and microglia consequently decreasing neurogenesis, it will be important to evaluate potential long-term consequences of infection in the adult brain. Postfetal infection with ZIKV will most certainly negatively impact the maintenance of neural homeostasis through aberrations in NSC and neuroinflammatory cell behavior. Thus, it seems likely that postfetal infection with ZIKV could result in the manifestation of neuropsychiatric and neurodegenerative disorders associated with disruptions in neurogenesis and chronic neural inflammation (see Table 1).

### Non-Traditional Model With High Natural Neuronal Turnover and Plasticity

Songbirds provide a powerful model for understanding the mechanisms, including those of the neuroimmune system, that regulate natural neuronal turnover during periods of homeostatic stability and robust plasticity. Avian song learning and production are regulated by discrete, well-characterized and intertwined circuits—the anterior forebrain pathway and song production pathway, respectively (Figure 3). The song production pathway includes the highly neurogenic nucleus HVC and its target nucleus, the robust nucleus of the archopallium (RA). In temporal resident and migratory (226) birds (most often the males), both HVC and RA undergo dramatic sex steroid dependent physiological and morphological changes between environmental seasons [reviewed in Ref. (227)]. Early each breeding season (spring) HVC and RA nearly double in volume. The increase in HVC volume results largely from the addition of over 50,000 new neurons to a pre-breeding season neuronal population of around 100,000 [numbers from Gambel’s white-crowned sparrows (*Zonotrichia leucophrys gambelii*) (228, 229) and vary across species (226)]. Alternatively, the growth of RA volume results from increases in neuron size and spacing, but not number (228, 229). Both HVC and RA demonstrate evidence of increased neural activity during the breeding season (230, 231), which has been proposed to be one function of the seasonal uptick in addition of new HVC neurons (81). The seasonal growth of the song production pathway nuclei is concomitantly linked to increased rate and quality of singing behavior (232), which is used most typically by male birds for territory maintenance and mate attraction. As seasonally plastic songbirds transition into the non breeding season (fall), plasmic levels of sex steroids decrease and the song production pathway regresses (89, 233, 234). Regression of HVC occurs very rapidly, with the caspase-mediated apoptosis of 50,000 neurons (a mixture of new and old neurons) occurring between one and four days following transition into non-breeding conditions (89, 235). As soon as one day following transition, song rate and quality also decreases dramatically (89).

Importantly, yet unsurprisingly given the evidence above, systemic inflammation negatively impacts hormonal responses and behavior in songbirds. Subcutaneous injection of LPS in the songbird Gambel’s white-crowned sparrow prompts a rapid increase in plasma corticosterone levels, suppresses luteinizing hormone (a trigger of gonad growth and production of sex steroids), and impairs total activity encompassing decreases in food and water intake, singing, and territorial aggression (236). These observations in conjunction with the known upregulation of aromatase expression by astroglia in the zebra finch telencephalon following injury (171, 201) firmly establish that inflammation impacts the avian CNS and likely plays a role in neural homeostasis and seasonal plasticity of the song circuits.

Interestingly, the seasonal regression of HVC through caspase-mediated apoptosis is necessary for reactive proliferation of neural stem/progenitor cells in the nearby VZ and subsequent HVC neuronal addition (89, 235, 237). Given the likely role of phagocytic cells in the clearance of dying HVC neurons, microglia, and astrocytes may confer the signal of neuronal death in HVC to the VZ neural stem/progenitor cells. If so, the potential for understanding the functions and mechanisms of microglia and astrocytes during natural death or turnover of neurons and the functional incorporation of new long-range projection neurons [as opposed to interneurons or locally projecting adult-born neurons in mammals (81, 104)] is unparalleled. However, to date, any functional mechanistic connections between sex steroid dependent neural plasticity, natural reactive neurogenesis, and neuroinflammatory involvement remains to be directly tested.

Songbirds also provide a tractable model for investigating the interactions between neurogenesis, neuroinflammation, and sex steroid levels, both elevated and basal in both males and females. Although all passerine songbirds have the neural circuits controlling song learning and production, not all songbirds are seasonally plastic. For example, zebra finches are highly social opportunistic breeders, and as such, do not have photoperiod-dependent seasonality of either systemic testosterone levels or HVC neuronal addition. Yet, even in non-seasonally plastic species of songbirds, new neurons do continuously enter and incorporate within HVC, replacing older HVC to RA projection neurons (110). Thus, comparative studies across species or manipulative studies in both seasonal and opportunistic species could provide mechanistic insight into the neuroimmune modulatory effects of sex steroids across the sexes and under varying reproductive states.

### Non-Traditional Model With High Regenerative Capacity

Teleost fishes have the most widespread and pronounced adult neurogenesis of any vertebrate examined, with 12–16 proliferative zone distributed across telencephalon, diencephalon, optic tectum, cerebellum, and hindbrain of fish [Figure 3; (83)]. Teleosts have indeterminate growth (i.e., continue to grow throughout life), and adult neurogenesis has been proposed to function not only in corresponding growth of the CNS but also in replacing naturally dying neurons [reviewed in Ref. (81, 238)]. The mechanisms of adult neurogenesis in teleost fish are characteristic of those in mammals and birds, with a few exceptions—for more on localization of proliferative zones and mechanisms, see Ref. (238).

Across all vertebrates traumatic brain injury is characterized by apoptosis of neurons, glia, and endothelial cells, inflammation, proliferation of micro- and macroglial cells, and increased neural stem/progenitor cell proliferation. Depending on the severity of the lesion, the secondary effects can include a penumbra of degeneration, inflammation, and breakdown of the BBB, all of which further complicate regenerative abilities and functional recovery of behavior. After traumatic brain injury, bony fishes have the remarkable ability to not only upregulate the proliferation of NSCs and migration of neuroblasts, but to functionally incorporate new neurons that survive long term and to near completely restore tissue architecture at the site of the lesion [reviewed in Ref. (239)]. The first evidence of such brain regeneration in fish came from optic tectum lesion experiments in juvenile carp (*Carassium carassium*), in which the size of the remaining progenitor zone correlated with the degree of tissue restoration (240). Reactive proliferation also occurs in the cerebellum of weakly electric fish (*Apteronotus leptorhynchus*) as soon as one day following apoptosis-mediated clearance of cells at the lesion site (241, 242). Apoptosis rather than necrosis at the lesion promotes a “clean” type of cell death, which has been proposed to contribute to the remarkable regenerative ability of fishes (241). Likewise, zebrafish exhibit reactive progenitor cell proliferation, neuroblast migration, nascent neuronal incorporation, and restoration of tissue architecture following stab lesions through the olfactory bulb and into the telencephalon (243).

Many factors regulate teleost reactive neurogenesis [reviewed in Ref. (239)], including inflammation. Traumatic injury to the zebrafish telencephalon stimulates the activation of microglia, infiltration of leukocytes, and the secretion of pro-inflammatory cytokines TNFα, IL-1β, and IL-8 (244). Upregulation of cysteinyl leukotriene receptor 1 expression in the NPC niche is also induced by traumatic brain injury as wells as cerebroventricular microinjection of the immunogenic zymosan A BioParticles (244). Moreover, administration of leukotriene C4, a ligand for CysLT1, enhances NPC proliferation without physical injury, suggesting cysteinyl leukotriene signaling is an essential regulator of the inflammatory-mediated neurogenic response in zebrafish (244). The chemokine receptor CXCR5 is expressed within and is a critical regulatory signal for the proliferation of the radial neural progenitors of the zebrafish telencephalon following stab lesion (245).

The profound regenerative ability of the fish CNS begs the question, “why is regeneration relatively limited in the mammalian brain?” Comparative studies uncovering the conserved and evolutionarily distinct molecular mechanisms that govern regenerative neurogenesis will inform not only neuroimmune and regenerative biology generally but also neurological disease etiology, and treatments that enhance NSC activity and successful neuroblast incorporation and survival.

## Concluding Remarks

Although still not extensively examined, there clearly exist important links between sex steroids, neuroinflammatory responses, and adult neurogenesis. In this review, I have provided convincing evidence that sex steroids independently influence the processes and mechanisms of adult neurogenesis and neuroimmune responses. Moreover, sex steroids are secreted as an effector of the inflammatory response, and so likely influence immune cell-mediated homeostatic neurogenesis and neural repair. Integration of the sub-fields of sex steroids, neuroinflammation, and neurogenesis will embolden a better understanding of basic biology of NSCs and neuroimmune function, as well as the etiology of neural disorders—those with established and emerging evidence of a neuroimmune component. Ultimately, future studies exploiting the intersection of sex steroids, inflammation, and neurogenesis will spur the development of novel therapeutic strategies for the treatment of neuropsychiatric and neurodegenerative disorders. The future of neuroinflammation and adult neurogenesis research will no doubt be complicated, but will also certainly be exciting and rewarding!

## Author Contributions

The author confirms being the sole contributor of this work and approved it for publication.

## Conflict of Interest Statement

The author declares that the research was conducted in the absence of any commercial or financial relationships that could be construed as a potential conflict of interest.
